# Peptide-Based Membrane Fusion Inhibitors Targeting HCoV-229E Spike Protein HR1 and HR2 Domains

**DOI:** 10.3390/ijms19020487

**Published:** 2018-02-06

**Authors:** Shuai Xia, Wei Xu, Qian Wang, Cong Wang, Chen Hua, Weihua Li, Lu Lu, Shibo Jiang

**Affiliations:** 1 Key Laboratory of Medical Molecular Virology of MOE/MOH, School of Basic Medical Sciences & Shanghai Public Health Clinical Center, Fudan University, 130 Dong An Rd., Xuhui District, Shanghai 200032, China; 15111010053@fudan.edu.cn (S.X.); xuwei11@fudan.edu.cn (W.X.); wang_qian@fudan.edu.cn (Q.W.); 16111010068@fudan.edu.cn (C.W.); 16211010047@fudan.edu.cn (C.H.); lul@fudan.edu.cn (L.L.); 2 Key Laboratory of Reproduction Regulation of National Population and Family Planning Commission, The Shanghai Institute of Planned Parenthood Research, Institute of Reproduction and Development, Fudan University, Shanghai 200032, China; weihua.li@sippr.org.cn; 3 Lindsley F. Kimball Research Institute, New York Blood Center, New York, NY 10065, USA

**Keywords:** HCoV-229E, membrane fusion, cell-cell fusion, peptide, inhibitor

## Abstract

Human coronavirus 229E (HCoV-229E) infection in infants, elderly people, and immunocompromised patients can cause severe disease, thus calling for the development of effective and safe therapeutics to treat it. Here we reported the design, synthesis and characterization of two peptide-based membrane fusion inhibitors targeting HCoV-229E spike protein heptad repeat 1 (HR1) and heptad repeat 2 (HR2) domains, 229E-HR1P and 229E-HR2P, respectively. We found that 229E-HR1P and 229E-HR2P could interact to form a stable six-helix bundle and inhibit HCoV-229E spike protein-mediated cell-cell fusion with IC_50_ of 5.7 and 0.3 µM, respectively. 229E-HR2P effectively inhibited pseudotyped and live HCoV-229E infection with IC_50_ of 0.5 and 1.7 µM, respectively. In a mouse model, 229E-HR2P administered intranasally could widely distribute in the upper and lower respiratory tracts and maintain its fusion-inhibitory activity. Therefore, 229E-HR2P is a promising candidate for further development as an antiviral agent for the treatment and prevention of HCoV-229E infection.

## 1. Introduction

Coronaviruses (CoVs) are positive-sense RNA enveloped viruses with various hosts, including avian species and mammals [1]. Currently, six CoVs have been reported to infect humans, including human coronavirus 229E (HCoV-229E), OC43 (HCoV-OC43), NL63 (HCoV-NL63), HKU1 (HCoV-HKU1), severe acute respiratory syndrome (SARS) coronavirus (SARS-CoV), and Middle East respiratory syndrome (MERS) coronavirus (MERS-CoV) [2,3,4,5]. During the 2002–2003 epidemic of SARS, approximately 8000 individuals were infected by SARS-CoV with a mortality rate of 10% [6]. During the 2012–2017 epidemic of MERS, 2121 laboratory-confirmed cases, including 740 related deaths (~35% mortality rate) in 27 countries, were reported to World Health Organization (WHO) [7]. Infection by other human coronaviruses, including HCoV-229E, HCoV-OC43, HCoV-NL63, and HCoV-HKU1, in healthy adults generally results in common cold with clinical manifestations that include discomfort, headache, sneezing, fever and cough. However, infection with these viruses in infants, elderly people, and immunocompromised patients may also cause severe diseases [2,8,9,10,11]. 

Although HCoV-229E was first isolated in 1966, molecular clock analyses indicated that it emerged about 200 years ago [12]. Evolutionary study suggested that its evolutionary origin was in hipposiderid bats and that camelids may serve as intermediate hosts [13]. Currently, HCoV-229E continues to evolve by mutation and recombination, leading to changes in its spike immunogenicity, infection mechanism, and even clinical symptoms [14,15,16]. For example, serological studies reported that infection of Sendai-H HCoV-229E, a new isolate, was possibly related to Kawasaki disease [16]. Therefore, it is essential to develop a potent inhibitor to treat HCoV-229E infection and control its potential epidemic spread.

**Figure 1:**
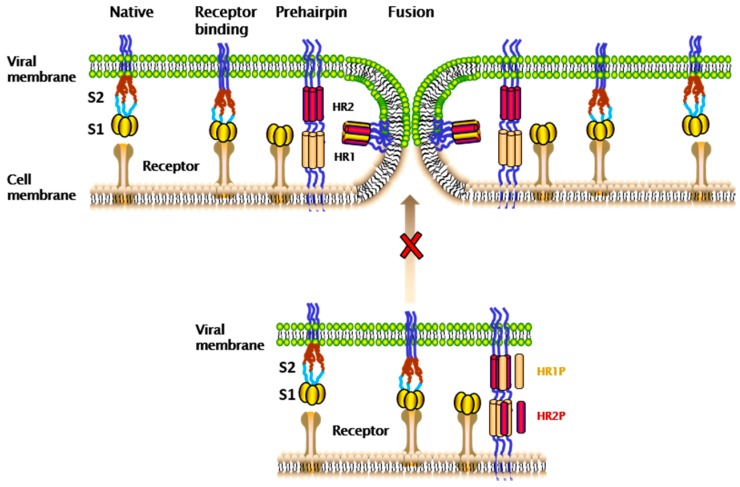
Model of membrane fusion mediated by coronavirus (CoV) spike (S) protein and the mechanism of heptad repeat 1 peptide (HR1P) and heptad repeat 2 peptide (HR2P). In the native state, the fusion peptide (FP), heptad repeat 1 (HR1) and heptad repeat 2 (HR2) domains in the S2 subunit are shielded by the S1 subunit. In the receptor-binding state, the S1 subunit binds with receptor on the target cell surface. In the pre-hairpin state, the S1 subunit dissociates, and FP in the S2 subunit is inserted into the target cell membrane, resulting in formation of the 6-HB fusion core by HR1 and HR2 and final fusion between the cell and virus membranes. HR1P and HR2P peptides can interact with the viral HR2 and HR1 domains, respectively, to block viral fusion core (6-HB) formation and inhibit viral and cellular membrane fusion. The “cross” and “arrow” means that the peptide HR1P or HR2P can block the viral gp41 6-HB-mediated membrane fusion process.

Similar to other CoVs, HCoV-229E belongs to type I enveloped virus. Its spike (S), consisting of S1 and S2 subunits on viral membrane, plays vital roles in viral entry into the host cells [2]. The S1 subunit contains the receptor-binding domain (RBD) for binding the cellular receptor, aminopeptidase N (APN) [17]. The S2 subunit, which is comprised of fusion peptide (FP), heptad repeat 1 (HR1), heptad repeat 2 (HR2), transmembrane domain (TM) and cytoplasmic domain fusion (CP), is responsible for mediating viral fusion and entry [18]. In the native state, HCoV-229E S protein is an inactive precursor [2]. During the viral infection process, target cell proteases can activate S protein by cleaving it into S1 and S2 subunits. For example, when HCoV-229E enters the host cell through the plasma membrane fusion pathway, HCoV-229E S protein can be cleaved and activated by transmembrane protease, serine 2 (TMPRSS2) and human airway trypsin-like protease (HAT) [2]. Interestingly, TMPRSS2 is expressed widely in the human respiratory tract where epithelial cells are also targeted for viral infection [2]. Therefore, TMPRSS2 plays an important role in promoting HCoV-229E infection in the human airway [2,14]. However, if HCoV-229E infects target cells through the endosomal membrane fusion pathway, then its S protein can be cleaved and activated by cathepsin L (CPL), a pH-dependent cysteine protease in endosome [19]. Previously, we and others discovered that C-peptides derived from the human immunodeficiency virus type 1 (HIV-1) gp41 HR2 region possess highly potent HIV-1 fusion inhibitory activities [20,21,22]. Among them, T20, was approved by the U.S. Food and Drug Administration (FDA) as the first HIV fusion inhibitor-based anti-HIV drug [23]. Subsequently, we identified several HR2-peptides targeting the HR1 domains of the S proteins of SARS-CoV and MERS-CoV with potent viral fusion inhibitory activity [24,25,26]. Their mechanisms of action have been well documented. As shown in Figure 1, after RBD binding of coronavirus S1 to the corresponding receptors, S2 changes conformation, including the insertion of FP into the target cell membrane, exposure of the pre-hairpin coiled coil of HR1 domain, and the interaction between viral HR2 domain and HR1 trimer to form six-helix bundle (6-HB), thus bringing the viral envelope and cell membrane into close proximity for viral fusion and entry. At the prefusion intermediate state, the peptides derived from the HR2 and HR1 domains of the CoV S proteins, designated HR2P and HR1P, interact with the viral exposed HR1-trimer and HR2 domain, respectively, and competitively inhibit viral autologous 6-HB formation, thus blocking virus-cell membrane fusion [27,28].

Although the HCoV-229E fusion core was characterized in 2006 [18], no peptide-based HCoV-229E fusion inhibitor has ever been reported. Therefore, we designed and synthesized peptides derived from the HR1 and HR2 domains of the HCoV-229E S protein S2 subunit and tested their inhibitory activity against HCoV-229E S protein-mediated cell-cell fusion and both pseudotyped and live HCoV-229E infection in the present study.

## 2. Results

### 2.1. Design and Biophysical Characterization of 229E-HR1P and 229E-HR2P

**Figure 2:**
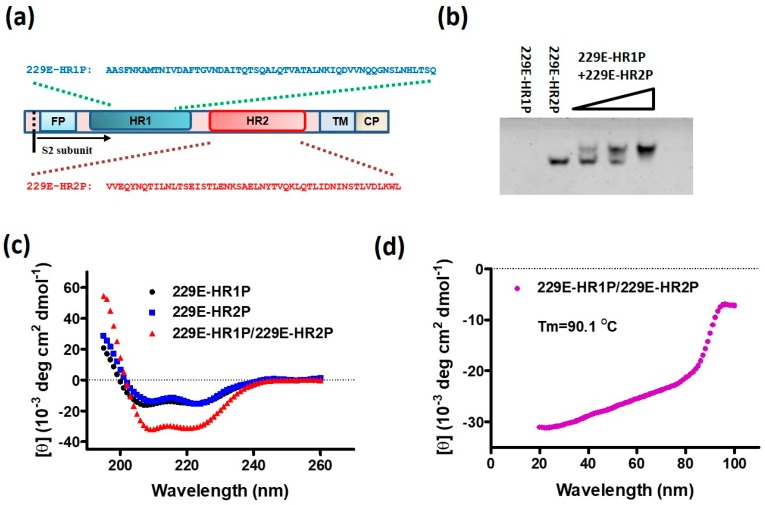
Design and biophysical characterization of 229E-HR1P and 229E-HR2P. (**a**) Schematic representation of HCoV-229E S protein S2 subunit, fusion peptide (FP), heptad repeat 1 (HR1), heptad repeat 2 (HR2), transmembrane domain (TM) and cytoplasmic domain (CP). 229E-HR1P and 229E-HR2P, derived from HR1 and HR2 domains, respectively, and their sequences are shown in the diagram; (**b**) Determination of the interaction of 229E-HR1P at 40 µM with 229E-HR2P at the concentrations of 20, 40, and 80 µM, respectively, by N-PAGE. 6-HB: six-helix bundle; (**c**) circular-dichroism (CD) spectra for 229E-HR1P, 229E-HR2P and their complex in phosphate buffer (pH 7.2); (**d**) Melting curves of the complex formed by 229E-HR1P and 229E-HR2P.

The fusion core in spike proteins of several coronaviruses have already been crystallized, and their related peptide fusion inhibitors have been studied [24,25,29]. However, HCoV-229E-specific fusion inhibitors have not been reported so far. Therefore, we herein report the design and synthesis of two peptides, designated 229E-HR1P and 229E-HR2P, spanning residues 795–850 in the HR1 domain and 1053–1102 in the HR2 domain of HCoV-229E spike protein, respectively (Figure 2a). 

To investigate whether 229E-HR1P and 229E-HR2P interact to form 6-HB, we incubated 229E-HR1P in phosphate buffer saline (PBS) at 40 μM with 229E-HR2P at 20, 40, and 80 μM, respectively, at 37 °C for 30 min before loading the samples to the Tris-glycine gel, followed by analysis with N-PAGE as previously described [25]. As shown in Figure 2b, similar to the HR1 peptides from SARS-CoV and MERS-CoV S proteins [24,25], 229E-HR1P alone displayed no band in the gel (lane 1) because this peptide carries net positive charges, thus moving up and off the gel under the native electrophoresis condition. 229E-HR2P alone showed a band at the lower part in the gel (lane 2). However, the mixtures of 229E-HR1P at 40 μM and 229E-HR2P at 20, 40 and 80 μM, respectively, showed new bands corresponding to the 6-HB at the upper part in the gel (lanes 3, 4 and 5), confirming that 229E-HR1P and 229E-HR2P do interact with each other to form 6-HB. 

To further study characteristics of the interaction between 229E-HR1P and 229E-HR2P, we determined the secondary structures of 229E-HR1P, 229E-HR2P and their complex in mixture (229E-HR1P/229E-HR2P) by circular-dichroism (CD) spectroscopy. As shown in Figure 2c, 229E-HR1P or 229E-HR2P alone displayed a relatively low α-helicity structure, whereas the mixture of 229E-HR1P/229E-HR2P at equimolar concentration showed a complex with high α-helicity, as characterized by the saddle-shaped negative peak at 208 and 222 nm in the far UV region of the CD spectrum. Meanwhile, this helical bundle showed strong thermal stability with a Tm of 90.1 °C (Figure 2d), which is higher than that of the MERS-CoV HR1P/HR2P complex (~87 °C). The above results confirm that peptides derived from HCoV-229E HR1 and HR2 domains are capable of harboring the biological activity required to mimic viral fusion core structure by forming 6-HB in vitro. Meanwhile, the 229E-HR1P/229E-HR2P complex provides a useful model for studying the detailed role of the HCoV-229E 6-HB fusion core in mediating viral fusion and entry.

### 2.2. Establishment of HCoV-229E S Protein-Mediated Cell-Cell Fusion Assay

**Figure 3:**
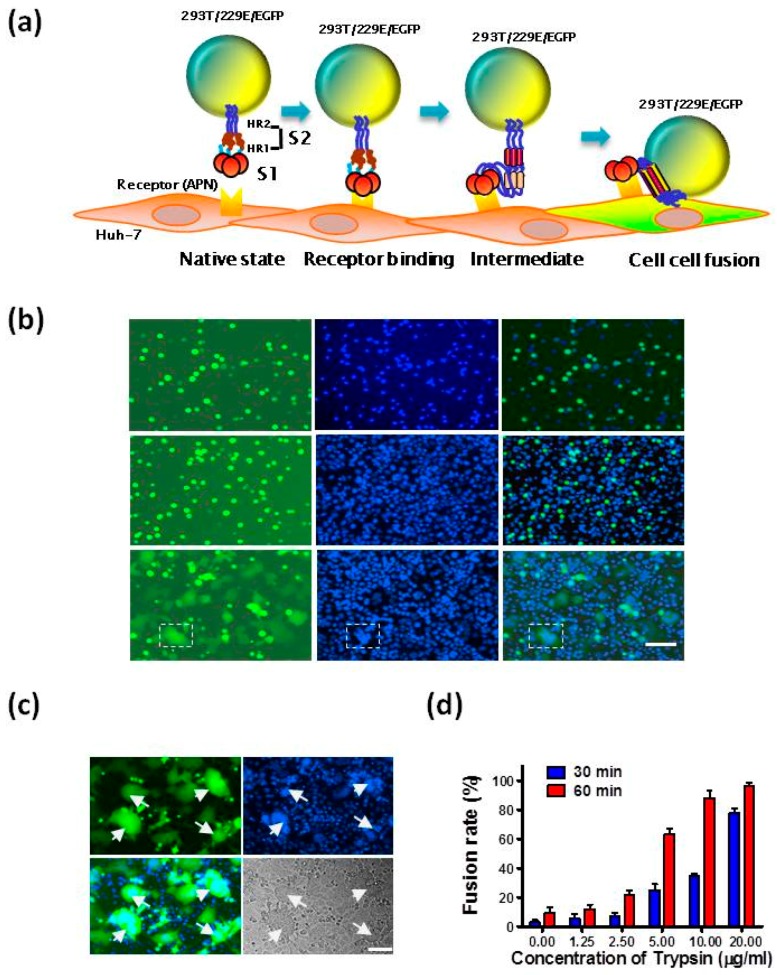
Establishment of HCoV-229E S protein-mediated cell-cell fusion assay. (**a**) Schematic representation of HCoV-229E S protein-mediated cell-cell fusion. 293T cells expressing the S protein on cellular surface and Enhanced Green Fluorescent Protein (EGFP) in cytoplasm (293T/MERS/EGFP) and Huh-7 cells expressing the receptor aminopeptidase N (APN) were used as the effector and target cells, respectively; (**b**) 293T/MERS/EGFP cells alone (upper), Huh-7 cells and 293T/EGFP (middle) or Huh-7 cells and 293T/MERS/EGFP cells (lower) were cultured or cocultured at 37 °C for 3–6 h, stained with 4′,6-diamidino-2-phenylindole (DAPI), and then photographed under an optical microscope with fluorescence (left) and DAPI (middle). The figures in left and middle panels were overlapped (right). The area encircled by the white dotted line is the fused cell; (**c**) After coculture at 37 °C for 24 h, the fused cells were photographed under fluorescence microscopy (upper left) and DAPI (upper right). The overlap picture (bottom left) shows that each of the fused cells contains two or more nuclei. The bottom right image was photographed under visible light. The arrows point to the fused cells; (**d**) The fusion rate of HCoV-229E S-mediated cell-cell fusion in the absence and presence of trypsin at different concentrations. Data are means ± standard deviation (SD) of triplicate samples from a representative experiment. Scale bars, 800 µm.

It has been reported that HCoV-229E-infected cells could form syncytia with uninfected cells in the presence of trypsin [19], suggesting, in turn, that the S protein on HCoV-229E-infected cells may mediate the fusion between infected and uninfected cells. Accordingly, to assess the inhibitory activities of 229E-HR1P and 229E-HR2P, we established a cell-cell fusion model. To accomplish this, we used Huh-7 cells, naturally expressing the APN receptor of HCoV-229E on the cell surface [17,30], and 293T cells that simultaneously coexpress HCoV-229E S protein on the cell surface and Enhanced Green Fluorescent Protein (EGFP) in the cytoplasm (293T/229E/EGFP) as the target and effector cells, respectively (Figure 3a). After coculture of effector cells (293T/229E/EGFP) and target cells (Huh-7) at 37 °C for 3 to 6 h, we could observe cells that had apparently fused, using fluorescence microscopy, which showed larger size and weaker fluorescence than unfused cells. This can be explained by the diffusion of fluorescence into one or more cells that fuse together (Figure 3b). After staining with 4′,6-diamidino-2-phenylindole (DAPI), we could clearly see several nuclei in one fused cell. However, each of the 293T/229E/EGFP cells cultured alone contained only one single nucleus, suggesting that the effector cells cannot spontaneously fuse with each other or do not undergo propagating divisions to influence the assessment of fusion. Besides, 293T/EGFP cells that express no HCoV-229E S protein on cell surface could not fuse with the target cells, and each of these cells also contained only one nucleus. After 24 h, obvious formation of syncytium occurred, as shown in Figure 3c. Consistent with the previous report [19], trypsin could effectively promote cell-cell fusion in a time- and dose-dependent manner (Figure 3d). This finding confirms that HCoV-229E S protein can mediate membrane fusion between effector cells and target cells, followed by syncytium formation, suggesting that this cell-cell fusion assay can be used to study HCoV-229E S protein-mediated membrane fusion process and identify HCoV-229E fusion and entry inhibitors.

### 2.3. HCoV-229E HR1P and HR2P Peptides Inhibited HCoV-229E S Protein-Mediated Cell-Cell Fusion

**Figure 4:**
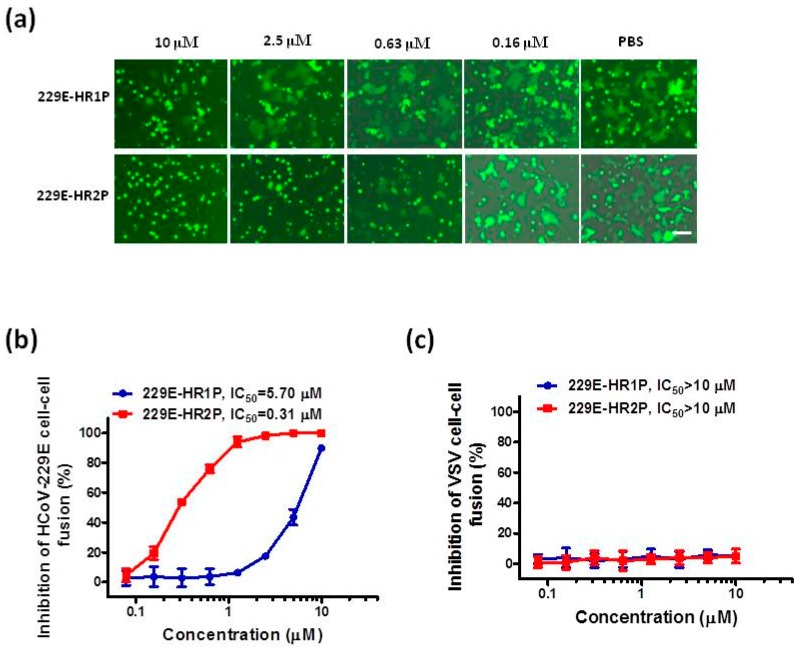
Inhibitory activities of 229E-HR1P and 229E-HR2P on cell-cell fusion mediated by HCoV-229E S protein or vesicular stomatitis virus (VSV) G protein. (**a**) Images of 229E S protein-mediated cell-cell fusion in the presence of 229E-HR1P (upper) and 229E-HR2P (lower) at different concentrations; (**b**) Inhibitory activities of 229E-HP1P and 229E-HR2P against HCoV-229E S-mediated cell-cell fusion; (**c**) Inhibitory activities of 229E-HP1P and 229E-HR2P on VSV G-mediated cell-cell fusion. Data are means ± SD of triplicate samples from a representative experiment. Scale bars, 800 µm.

Next, we used our established cell-cell fusion assay described above to test the inhibitory activities of 229E-HR1P and 229E-HR2P on HCoV-229E S-mediated cell-cell fusion. As shown in Figure 4, 229E-HR1P and 229E-HR2P could effectively inhibit HCoV-229E S-mediated cell-cell fusion with IC_50_ of 5.7 and 0.3 μM, respectively, while they exhibited no significant inhibition of vesicular stomatitis virus (VSV) G protein-mediated cell-cell fusion at the concentration as high as 10 μM. Together, these results suggested that 229E-HR2P is more effective than 229E-HR1P in inhibiting HCoV-229E S-mediated membrane fusion.

### 2.4. HCoV-229E-HR1P and -HR2P Peptides Inhibited Infection of Pseudotyped and Live HCoV-229E

**Figure 5:**
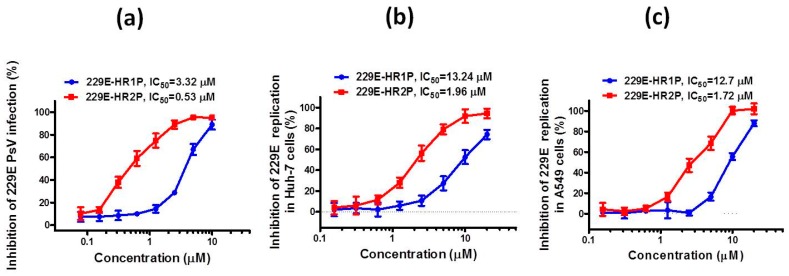
Inhibitory activity of 229E-HR1P and 229E-HR2P against pseudotyped and live HCoV-229E infection. (**a**) 229E-HR1P- and 229E-HR2P-HCoV-229E pseudovirus (PsV) infection in Huh-7 cells; (**b**) 229E-HR1P- and 229E-HR2P-mediated inhibition of HCoV-229E infection and replication in Huh-7 cells; (**c**) 229E-HR1P- and 229E-HR2P-mediated inhibition of HCoV-229E infection and replication in A549 cells. Data are means ± SD of triplicate samples from a representative experiment.

We then used our established HCoV-229E pseudovirus infection assay to test the potential inhibitory activities of 229E-HR1P and 229E-HR2P on HCoV-229E pseudovirus infection in Huh-7 cells. As shown in Figure 5a, 229E-HR2P could significantly inhibit HCoV-229E pseudovirus infection in a dose-dependent manner, with an IC_50_ value of 0.5 μM, whereas 229E-HR1P exhibited only moderate inhibition with an IC_50_ of 3.3 μM. These results further confirm that 229E-HR2P is more effective than 229E-HR1P in inhibiting HCoV-229E pseudovirus infection, consistent with previous studies reporting on the antiviral activities of peptide-based SARS-CoV and MERS-CoV fusion inhibitors [24,25].

Next, we used our established cytopathic effect (CPE)-based viral inhibition assay to determine the antiviral activities of 229E-HR1P and 229E-HR2P against live HCoV-229E infection and replication in Huh-7 and A549 cells. We found that 229E-HR1P and 229E-HR2P could effectively inhibit HCoV-229E infection and replication in Huh-7 cells in a dose-dependent manner with IC_50_ values of 13.2 μM and 1.96 μM, respectively (Figure 5b), consistent with their inhibitory activities in A549 cells, which is a human respiratory epithelial cell line and susceptible to HCoV-229E infection and replication (Figure 5c) [30].

### 2.5. HCoV-229E-HR1P and -HR2P Peptides Lacked Cytotoxicity to Huh-7 and A459 Cells

**Figure 6:**
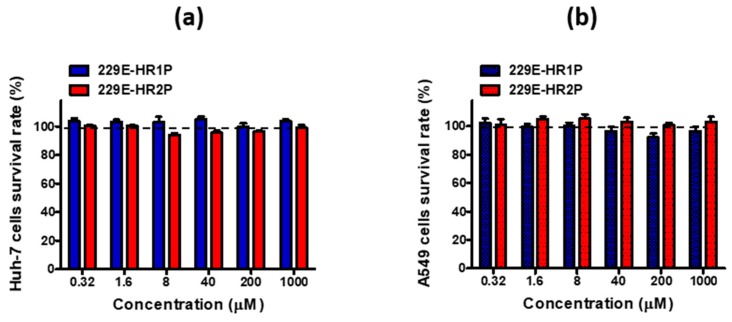
Cytotoxicity of 229E-HR1P and 229E-HR2P. (**a**) Cytotoxicity of 229E-HR1P and 229E-HR2P to Huh-7 cells; (**b**) Cytotoxicity of 229E-HR1P and 229E-HR2P to A549 cells. Data are means ± SD of triplicate samples from a representative experiment.

We then tested the potential cytotoxicity of 229E-HR1P and 229E-HR2P on Huh-7 and A549 cells, which were used as target cells in assays for cell-cell fusion, both pseudotyped and live HCoV-229E infection. Neither 229E-HR1P nor 229E-HR2P had significant cytotoxicity to these cells at concentrations up to 1,000 μM (Figure 6). The selectivity index (SI: CC_50_/IC_50_) of 229E-HR2P was more than 500, suggesting it could be further developed as an effective and safe HCoV-229E fusion inhibitor for clinical use.

### 2.6. 229E-HR2P Retained Its Fusion-Inhibitory Activity in Mouse Upper and Lower Respiratory Tract

**Figure 7:**
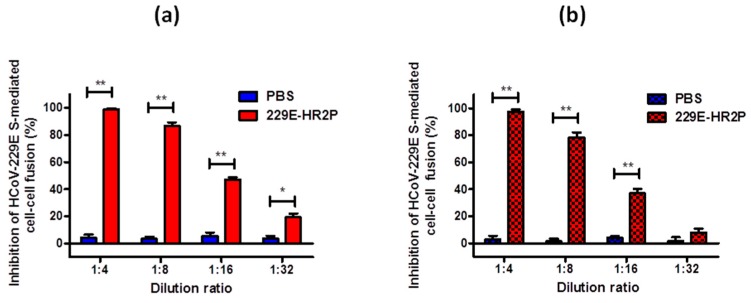
Inhibitory activity of 229E-HR2P in mouse upper and lower respiratory tracts against HCoV-229E S-mediated cell-cell fusion. (**a**) Fusion inhibitory activity of upper respiratory tract lavage fluid of mice treated with or without 229E-HR2P; (**b**) Fusion inhibitory activity of lower respiratory tract lavage fluid of mice treated with or without 229E-HR2P. Data are means ± SD of triplicate samples from a representative experiment. The asterisks represent significant differences: ** *p* < 0.01; * *p* < 0.05.

The respiratory tract is a key target of HCoV-229E viral infection [14]. Proteolytic enzymes on the surface of respiratory tract mucosa may cause degradation of the peptides [31,32], resulting in attenuation of their antiviral activity. Therefore, the antiviral activity of 229E-HR2P was verified in the respiratory tract of mice intranasally administered with or without 229E-HR2P. One hour later, upper and lower respiratory tract lavage fluids, respectively, were collected to test their inhibitory activities on HCoV-229E S protein-mediated cell-cell fusion. As shown in Figure 7, the upper and lower respiratory tract lavage fluids from mice treated with 229E-HR2P at 1:32 and 1:16 dilutions, respectively, exhibited significant viral membrane fusion-inhibitory activity. These findings suggested that 229E-HR2P may not be degraded by proteolytic enzymes and that the peptide can maintain effective antiviral activity in both upper and lower respiratory tracts against HCoV-229E infection.

## 3. Discussion

Based on our previous experience in discovery of fusion inhibitory peptides against HIV [20], SARS-CoV [24] and MERS-CoV [25], we designed and synthesized peptides, 229E-HR1P and 229E-HR2P, derived from the HR1 and HR2 domains of the HCoV-229E S protein S2 subunit, respectively. Based on biophysical analyses, we have shown that these peptides interact to form 6-HB with strong thermal stability, suggesting that 229E-HR1P and 229E-HR2P may interact with the viral HR2 and HR1 domains, respectively, to form heterologous 6-HB, thus blocking viral fusion core formation and inhibiting fusion between the viral and target cell membranes. Indeed, 229E-HR2P showed high potency in inhibiting HCoV-229E S-mediated cell-cell fusion in both pseudotyped and live HCoV-229E infection, which is consistent with the results of HR2 peptides against HIV, SARS-CoV and MERS-CoV [20,24,25]. However, in contrast to the HR1 peptides derived from the SARS-CoV and MERS-CoV S proteins that show no membrane fusion inhibitory activities [24,25], 229E-HR1P also exhibited inhibitory against HCoV-229E infection, albeit with less potency than 229E-HR2P. This is possibly because HR1 peptide from HCoV-229E S protein has higher α-helicity than that from either SARS-CoV or MERS-CoV [24,25]. Therefore, it may be easier for HCoV-229E HR1P to form α-helical trimer to interact with the viral HR2 domain. Similarly, the N28 peptide derived from the HIV-1 gp41 HR1 domain could not form α-helical trimer in solution and thus lacked HIV-1 fusion inhibitory activity. However, the N28Fd, in which the trimerization motif Foldon (Fd) was conjugated to C-terminus of N28, could form α-helical trimer in solution and exhibit potent anti-HIV-1 activity [33]. Therefore, the antiviral activity of 229E-HR1P may be improved by adding Fd to the C-terminus of 229E-HR1P peptide.

Similar to other coronaviruses, HCoV-229E enters the target cell through two pathways, either plasma membrane fusion or endosomal membrane fusion, depending on the protease present on the target cells [2]. In the endosomal pathway, CPL plays an important role in cleaving and activating 229E S protein, and the inhibitors of CPL could inhibit HCoV-229E infection [19]. Bertram et al. have reported that TMPRSS2 on the target cells in the respiratory epithelium could cleave and activate 229E S to promote viral infection [2]. In human airway epithelial cells, TMPRSS2 is expressed on the cell surface and is associated with several coronavirus receptors, such as angiotensin converting enzyme 2 (ACE2), dipeptidyl peptidase-4 (DPP4), and APN, suggesting that TMPRSS2 can assist many coronaviruses to infect human airway epithelial cells through the plasma membrane fusion route [2,34,35,36]. Most recently, Matsuyama et al. have reported that the current clinical isolates of HCoV-229E preferably use TMPRSS2, rather than CPL, to infect epithelial cells in the human respiratory tract [14], suggesting that HCoV-229E-specific fusion inhibitory peptides are expected to inhibit membrane fusion mediated by HCoV-229E S protein that is proteolytically processed by TMPRSS2.

Different coronaviruses possess different infection characteristics in the human respiratory tract. For example, MERS-CoV mainly infects the human lower respiratory tract [37], while HCoV-229E is prone to infect the human upper respiratory tract, causing the common cold [38]. However, other studies have shown that HCoV-229E mainly causes lower respiratory tract infection in pediatric cases [39]. Here, we found that 229E-HR2P could retain its anti-HCoV-229E activity in both upper and lower respiratory tracts, suggesting that it can be used to block HCoV-229E infection in both upper and lower respiratory tracts by intranasal application.

Recently, several coronavirus inhibitors with different mechanisms of action against HCoV-229E infection in vitro have been reported. For example, Cinanserin targets viral 3C like (3CL) proteinase [40]; K22 inhibits the synthesis of membrane-bound viral RNA [41]; *N*-(2-hydroxypropyl)-3-trimethylammonium chitosan chloride (HTCC) polymers inhibit the interaction between virus and receptor [42]; Pyrrolidine-2 (Py-2) is a phospholipase A2α (PLA_2_α) inhibitor [43]; silvestrol is a eukaryotic initiation factor 4E (eIF4A) inhibitor [44]; and chloroquine inhibits the activation of cellular mitogen-activated protein kinases (MAPKs) [45]. In contrast to these inhibitors, 229E-HR1P and 229E-HR2P inhibit fusion and entry of HCoV-229E into the target cells by targeting HR2 or HR1, respectively. Therefore, it is reasonable to speculate that the combination of 229E-HR2P or 229E-HR1P and the above inhibitors with different antiviral mechanisms may have a synergistic effect against HCoV-229E infection. We have previously demonstrated that intranasal application of HR2PM2 and interferon-β (IFN-β) in combination exhibited significant synergistic anti-HCoV-229E activity [26].

In summary, we have designed and synthesized two HCoV-229E-specific peptide fusion inhibitors, 229E-HR1P and 229E-HR2P, derived from the HR1 and HR2 domains of HCoV-229E S protein S2 subunit. Both peptides can interact to form highly stable 6-HB. Between them, 229E-HR2P exhibited more potent inhibitory activity than 229E-HR1P against HCoV-229E S protein-mediated cell-cell fusion, HCoV-229E pesudovirus infection and live HCoV-229E replication in human respiratory epithelial cells with no detectable cytotoxicity. Meanwhile, 229E-HR2P is able to maintain its fusion-inhibitory activity in both upper and lower respiratory tracts, suggesting its promise as a candidate for development into an effective and safe HCoV-229E fusion inhibitor for prevention and treatment of HCoV-229E infection.

## 4. Materials and Methods

### 4.1. Cells and Viruses

293T, Huh-7, A549 and MRC-5 cells were cultured in Dulbecco’s modified Eagle’s medium (DMEM, Invitrogen, Carlsbad, CA, USA). All cell culture media were supplemented with 10% fetal bovine serum (FBS, Biowest, Loire Valley, France) and the antibiotics penicillin and streptomycin (Cytogen, Wetzlar, Germany). Cells were maintained at 37 °C under a 5% CO_2_ atmosphere. The American Type Culture Collection (ATCC) strain of HCoV-229E (VR-740) was propagated in MRC-5 cells. The viral titer (TCID_50_/mL) was determined on Huh-7 cells [30].

### 4.2. Peptides

Peptides were synthesized by KareBay Biochem (Ningbo, China), using a standard solid-phase 9-flurorenylmethoxycarbonyl (FMOC) method. All peptides were tested by high-performance liquid chromatography (HPLC) with purity >95%. The peptides were stored at −20 °C and solubilized with dimethyl sulfoxide (DMSO) or PBS. The concentrations of the peptides were tested by UV absorbance (Nanodrop2000, Thermo Fisher Scientific Inc., Waltham, MA, USA), and molar-extinction coefficient was calculated based on tryptophan and tyrosine residues [46].

### 4.3. Native Page

Native polyacrylamide gel electrophoresis (N-PAGE) was performed as previously described [25]. Tris-glycine gels (12%) and tricine glycine running buffer (pH 8.3) were used for N-PAGE. Peptide 229E-HR1P in PBS (40 μM) was incubated with peptide 229E-HR2P (20, 40, and 80 μM, respectively) at 37 °C for 30 min. Peptides were mixed with Tris-glycine native sample buffer and then loaded onto N-PAGE gel. Electrophoresis was performed at room temperature for approximately 2 h using a constant voltage of 125 V. The gel was stained with Coomassie blue and imaged with a FluorChem Imaging System (Alpha Innotech/ProteinSimple, San Jose, CA, USA).

### 4.4. Circular Dichroism (CD) Spectroscopy

Circular dichroism (CD) spectroscopy was performed according to our previously described protocols [25]. Briefly, 229E-HR1P was incubated with an equal molar concentration of 229E-HR2P at 37 °C for 30 min. The final concentration of each peptide was 10 μM in PBS (pH 7.2). CD spectra were tested on a spectropolarimeter (Model J-815; Jasco, Inc., Easton, MD, USA) from 195 to 260 nm. The baseline curve was determined on PBS alone. The ellipticity value of −3.3 × 10^−4^ deg·cm^2^·dmol^−1^ at 222 nm was taken as 100% helix formation [47,48]. A melting temperature (Tm) experiment was conducted by monitoring the change in ellipticity at 222 nm from 20 °C to 100 °C at a rate of 2 °C/min. 

### 4.5. HCoV-229E S Protein-Mediated Cell-Cell Fusion

Similar to our MERS-CoV cell-cell fusion system previously described [25], we cloned the 229E spike gene into the pAAV-IRES-EGFP vector and then transfected the recombinant plasmid pAAV-229E-IRES-EGFP into 293T cells (293T/229E/EGFP) to transiently express GFP protein in cytoplasm and 229E-S on the membrane surface. Herein we used 293T/229E/EGFP as effector cells and Huh-7 cells, which naturally express receptor APN on the membrane surface, as target cells. Then, 293T/229E/EGFP cells cultured alone or cocultured with Huh-7 cells at 37 °C for 3–6 h. After staining the cell nucleus with DAPI, the fused and unfused cells were visible under an optical microscope with fluorescence (Nikon, Tokyo, Japan) [26]. After further coculture at 37 °C for 24 h, syncytium formation was observed between 293T/229E/EGFP cells and Huh-7 cells. In addition to microscopy, 293T/229E/EGFP cells and Huh-7 cells were coincubated at 37 °C for 30 min or 1 h in the absence or presence of Trypsin with graded concentrations, and the number of fused and unfused cells was counted to calculate the fusion rate. Here, we used 293T cells expressing only EGFP (293T/EGFP) as effector cells for control.

### 4.6. Inhibition of Cell-Cell Fusion by Peptides

Effector cells (293T/VSV/EGFP) produced by cotransfecting plasmid carrying the sequence of the VSV-G protein and the pAAV-IRES-GFP vector were then fused with Huh-7 cells under the conditions as described elsewhere [49]. In the absence or presence of the detected peptides with different concentrations, Huh-7 cells were coincubated with 293T/229E/EGFP cells or 293T/VSV/EGFP cells at 37 °C for 3–6 h. The number of fused and unfused cells was counted, and the percentage inhibition of cell-cell fusion was calculated according to the previously described method [25].

### 4.7. Inhibition of HCoV-229E Pseudovirus Infection

Pseudovirus carrying HCoV-229E S protein was constructed, and the HCoV-229E pseudovirus infection assay was developed based on our experience in establishing the MERS-CoV pseudovirus infection assay performed as described previously [26,50]. Briefly, HCoV-229E pseudovirus was generated via cotransfection of 293T cells with an HCoV-229E S protein-expressing plasmid (pcDNA3.1-229E, kindly provided by Fang Li) and a backbone plasmid (pNL4.3-HIV-luc). Cell supernatants were harvested 48 h after transfection and centrifuged at 3000× *g* at 4 °C for 10 min. To measure the antiviral activities of peptides, peptides to be tested were prepared in 2-fold dilutions and incubated with pseudovirus for 1 h at 37 °C. The virus/peptide mixture was added to Huh-7 cells (10^4^ per well). The culture was refed with fresh medium 12 h post-infection and incubated for an additional 48 h at 37 °C. Luciferase activity was measured using luciferase assay reagents (Promega, Madison, WI, USA) and a luminescence counter (Infinite M200PRO, Tecan, NC, USA). The percent inhibition and IC_50_ values were calculated using the CalcuSyn software (kindly provided by T.C. Chou) [51]. 

### 4.8. Inhibition of HCoV-229E Replication

A CPE-based viral inhibition assay for measuring the antiviral activity of peptides against live HCoV-229E was established as we did for detecting anti-Zika virus activity [52]. Briefly, a 50 μL peptide solution was mixed with 50 μL of HCoV-229E (VR-740, 100 TCID_50_). After incubation at 37 °C for 1 h, the mixture was added to Huh-7 or A549 cells seeded in 96-well plates, followed by incubation at 37 °C for 12 h [53]. The culture supernatant was replaced with fresh and serum-free DMEM. Three to eight days later, when 229E-induced CPE became evident, antiviral activity was detected using the Cell Counting Kit-8 (CCK8, Dojindo, Kumamoto, Kyushu, Japan), according to the instruction manual [52]. Data were then collected by microplate reader (Tecan).

### 4.9. Cytotoxicity Assay

The cytotoxicity of peptides to Huh-7 and A549 cells was measured following the instructions in the manual provided in the Cell Counting Kit-8 (CCK-8; Dojindo, Kumamoto, Kyushu, Japan). Briefly, a series of dilutions of 229E-HR1P or 229E-HR2P were mixed with 10,000 target cells in each well of 96-well plates. After incubation for 48 h, the culture medium was replaced by 100 μL of fresh cell medium with 4 μL of the solution from CCK-8. After incubation for an additional 2 h at 37 °C, the absorbance at 450 nm (A_450_) was detected as the readout of cell viability [54].

### 4.10. Detection of 229E-S Mediated Cell-Cell Fusion-Inhibitory Activity of 229E-HR2P in Mouse Respiratory Tract

All animal experimental procedures were carried out according to ethical guidelines and approval by Institutional Laboratory Animal Care and Use Committee at Fudan University (approval number 20160927-1, approval date 27 September 2016). The antiviral activity of a peptide in the respiratory tract of mice intranasally administered was tested as previously described [55,56,57]. Briefly, eight-week-old female Balb/c mice were divided into two groups. Then each mouse was administered PBS (40 μL) with or without 229E-HR2P (0.5 mg) by the intranasal route, respectively. After 1 h, mice were sacrificed by euthanasia, the upper airways lavaged with 300 μL of PBS, and 1 mL of PBS used to flush the lungs three times to obtain lower. Lavage fluids were then plated in serial dilutions to detect their inhibitory activity on HCoV-229E S-mediated cell-cell fusion.
